# Bridging Digital Divides: GWEP Pivots to Support Telehealth for Clinical Care and Education

**DOI:** 10.1093/geroni/igab046.1835

**Published:** 2021-12-17

**Authors:** Jung-Ah Lee, Lisa Gibbs, Julie Rousseau, Sonia Sehgal, Neika Saville

**Affiliations:** 1 University of California, Irvine, Irvine, California, United States; 2 UC Irvine Health, UC Irvine Health, California, United States; 3 University of California, Irvine, Orange, California, United States; 4 Division of Geriatric Medicine and Gerontology, Orange, California, United States

## Abstract

Early in the pandemic, the University of California, Irvine (UCI), GWEP pivoted to focus on building telehealth and remote patient monitoring, while supporting team-based interdisciplinary learners. Our Health Assessment Program for Seniors (HAPS) adapted to provide hybrid remote/in-person evaluations with our Geriatric Fellows and Doctor of Nurse Practitioner (DNP) students working alongside our multi-disciplinary team. Learner teams innovatively bridged the digital divide through weekly DNP support phone calls, and the Fellows delivered family conferences through Zoom. In ASSIST, medical students and nursing students gained digital competencies through a phone support system for isolated older adults with friendly weekly check-ins providing referrals to community resources. Another IRB-approved pilot, Healing at Home, diverted patients from the Emergency Room and In-Patient care with a team of ED, Hospitalists, Geriatricians teaching DNP and Fellows telehealth management. GWEP successfully piloted symbiotic learning for both older adults and health profession students through new virtual formats.

